# Supplementary material for: Combined Treatment of Cancer Cells Using Allyl Palladium Complexes Bearing Purine-Based NHC Ligands and Molecules Targeting MicroRNAs miR-221-3p and miR-222-3p: Synergistic Effects on Apoptosis
Source: Pharmaceutics. 2023 Apr 24;15(5):1332. doi: 10.3390/pharmaceutics15051332 (PMC10222778; doi:10.3390/pharmaceutics15051332)
Supplement: Supplementary file 1 [file pharmaceutics-15-01332-s001.zip › pharmaceutics-2164652-supplementary.pdf]

pharmaceutics

MDPI

Article

Combined treatment of cancer cells using allyl palladium complexes bearing purine-based NHC ligands and molecules targeting microRNAs miR-221-3p and miR-222-3p: synergistic effects on apoptosis

Chiara Tupini[1†], Matteo Zurlo[1†], Jessica Gasparello[1], Irene Lodi[1], Alessia Finotti[1,2], Thomas Scattolin[3], Fabiano Visentin[4], Roberto Gambari[1,2*], and Ilaria Lampronti[1,2*]

[1] Department of Life Sciences and Biotechnology, Ferrara University, 44121 Ferrara, Italy; chiara.tupini@unife.it, matteo.zurlo@unife.it, irene01.lodi@student.unife.it, jessica.gasparello@unife.it, alessia.finotti@unife.it, gam@unife.it, lmi@unife.it

[2] Center of Innovative Therapies for Cystic Fibrosis (InnThera4CF) University of Ferrara, Italy; alessia.finotti@unife.it, gam@unife.it, lmi@unife.it

[3] Dipartimento di Scienze Chimiche, Università degli Studi di Padova, via Marzolo 1, 35131 Padova, Italy; thomas.scattolin@unive.it

[4] Dipartimento di Scienze Molecolari e Nanosistemi, Universit Ca' Foscari, CampusScientifico Via Torino 155, 30174 Venezia-Mestre, Italy; fvise@unive.it

* Authors to whom correspondence should be addressed.

† These authors contributed equally to this work.

Citation: To be added by editorial staff during production.

Academic Editor: Firstname Lastname

Received: date
Accepted: date
Published: date

Publisher's Note: MDPI stays neutral with regard to jurisdictional claims in published maps and institutional affiliations.

Copyright: © 2022 by the authors. Submitted for possible open access publication under the terms and conditions of the Creative Commons Attribution (CC BY) license (https://creativecommons.org/licenses/by/4.0/).

SUPPLEMENTARY MATERIALS

Supplementary Figures

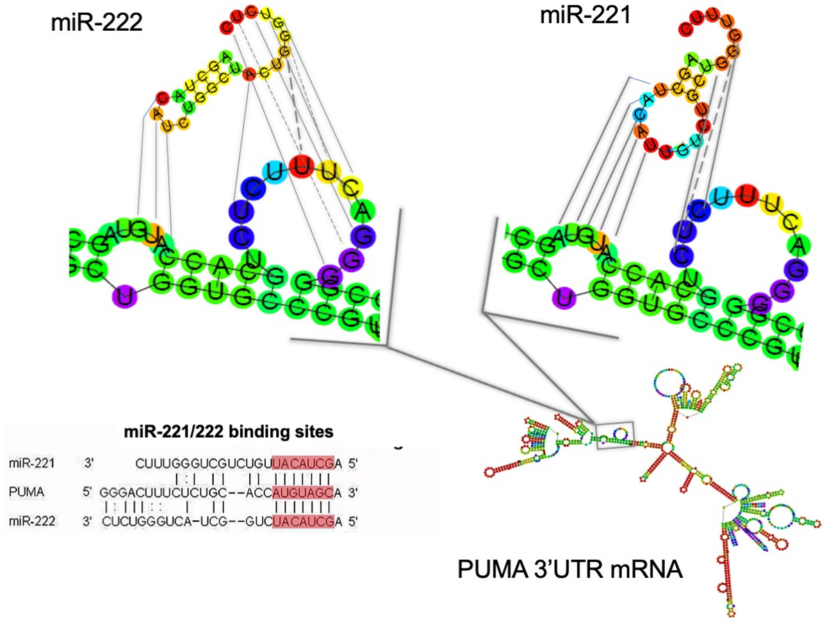

Figure S1 - Structures of miR-221, miR-222 and the 3′-UTR sequence of human PUMA mRNA, showing the interactions between miR-221/miR-222 miRNAs and the PUMA 3′-UTR mRNA. In the lower panel, the base-pairing between miR-221, miR-222 and the miR-221/222 PUMA binding site is shown.

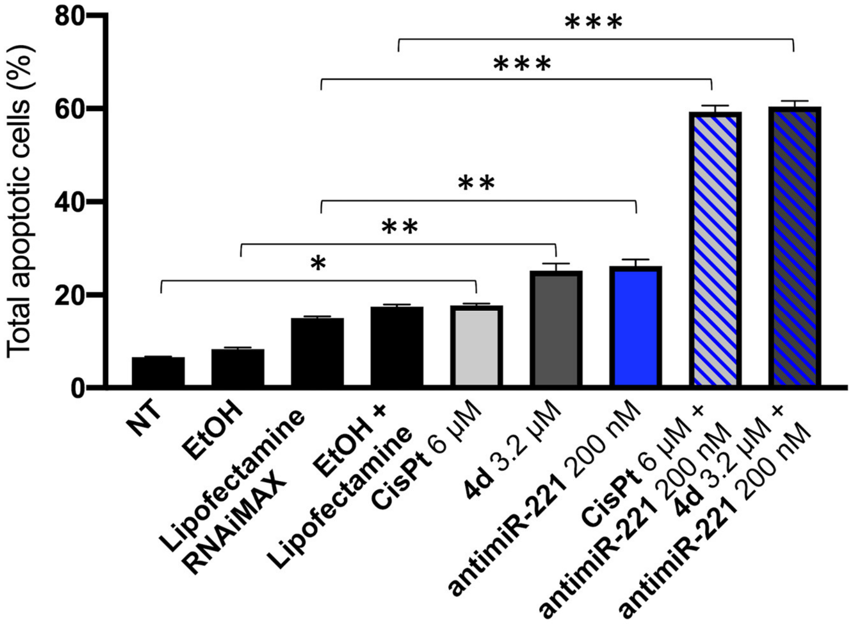

Figure S2. Total apoptotic cells percentage obtained from annexin-v staining after treatment of HT-29 cell line with CisPt and compound 4d in combination with antimir-221. Complete data set obtained from three different experiments, including each vehicle employed and showing comparisons evaluated for statistical analysis (ANOVA). $p < 0.05$ (*, significant), $p < 0.01$ (**; highly significant), $p < 0.001$ (***; highly significant).

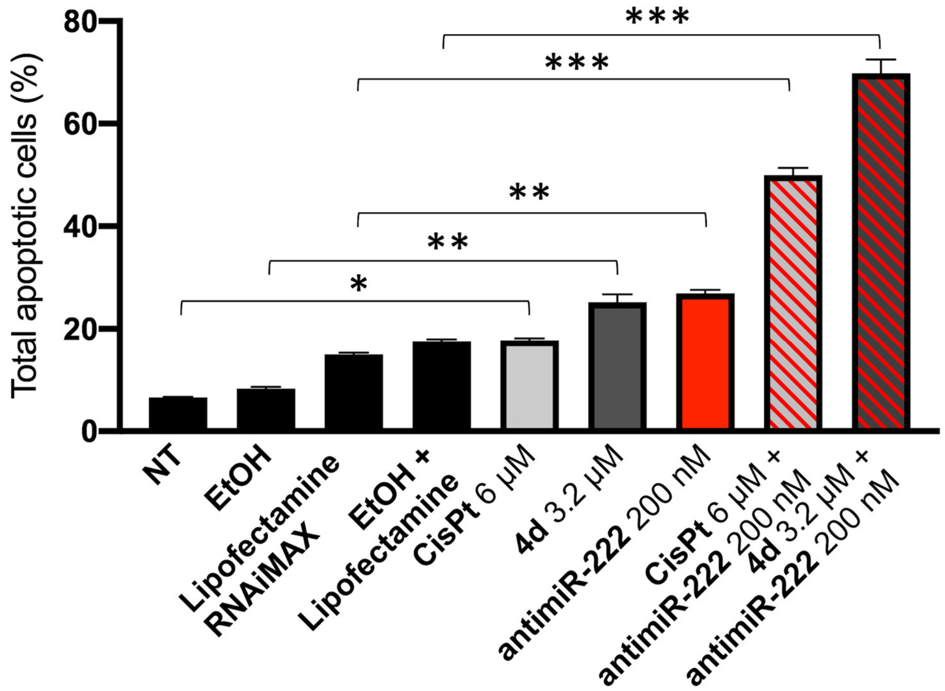

Figure S3. Total apoptotic cells percentage obtained from annexin-v staining after treatment of HT-29 cell line with CisPt and compound 4d in combination with antimir-222. Complete data set obtained from three different experiments, including each vehicle employed and showing comparisons evaluated for statistical analysis (ANOVA). $p < 0.05$ (*, significant), $p < 0.01$ (**; highly significant), $p < 0.001$ (***; highly significant).

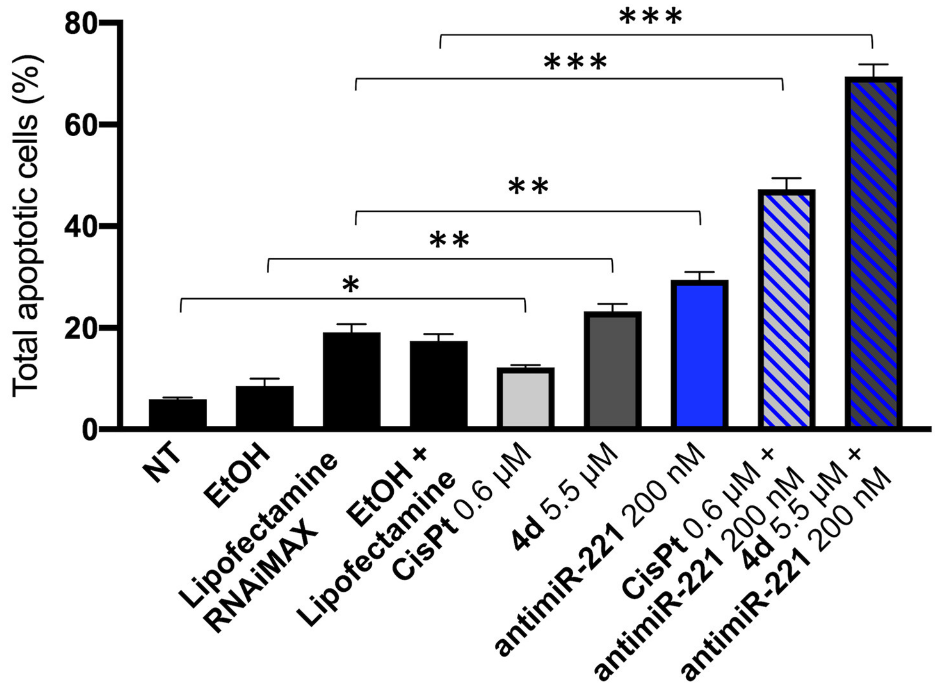

Figure S4. Total apoptotic cells percentage obtained from annexin-v staining after treatment of U251 cell line with CisPt and compound 4d in combination with antimir-221. Complete data set obtained from three different experiments, including each vehicle

employed and showing comparisons evaluated for statistical analysis (ANOVA). $p < 0.05$ (*, significant), $p < 0.01$ (**; highly significant), $p < 0.001$ (***; highly significant).

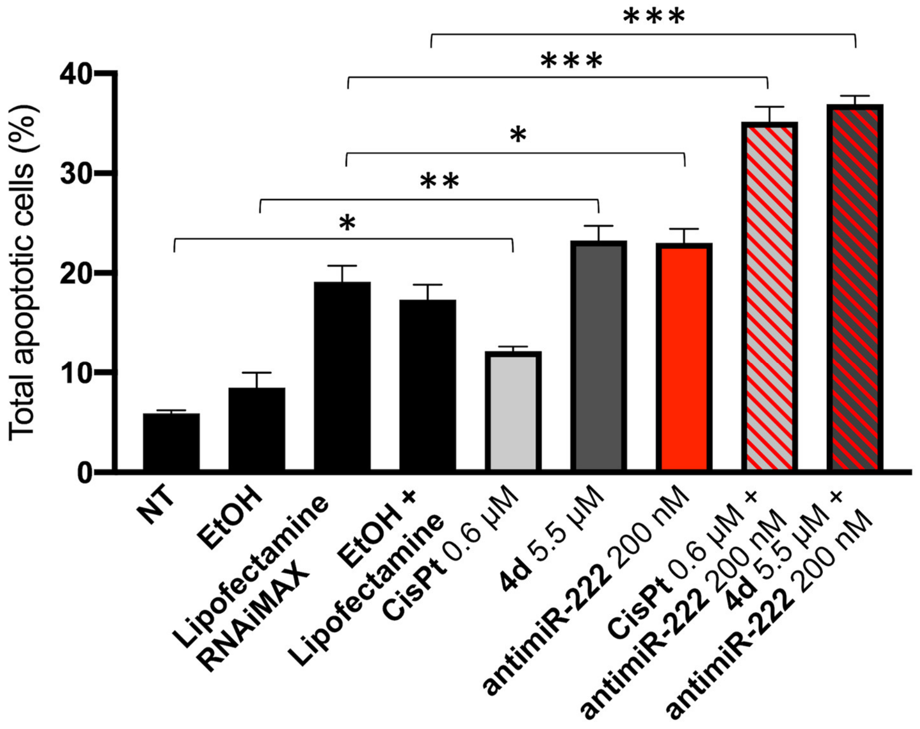

Figure S5. Total apoptotic cells percentage obtained from annexin-v staining after treatment of U251 cell line with CisPt and compound 4d in combination with antimir-222. Complete data set obtained from three different experiments, including each vehicle employed and showing comparisons evaluated for statistical analysis (ANOVA). $p < 0.05$ (*, significant), $p < 0.01$ (**; highly significant), $p < 0.001$ (***; highly significant).

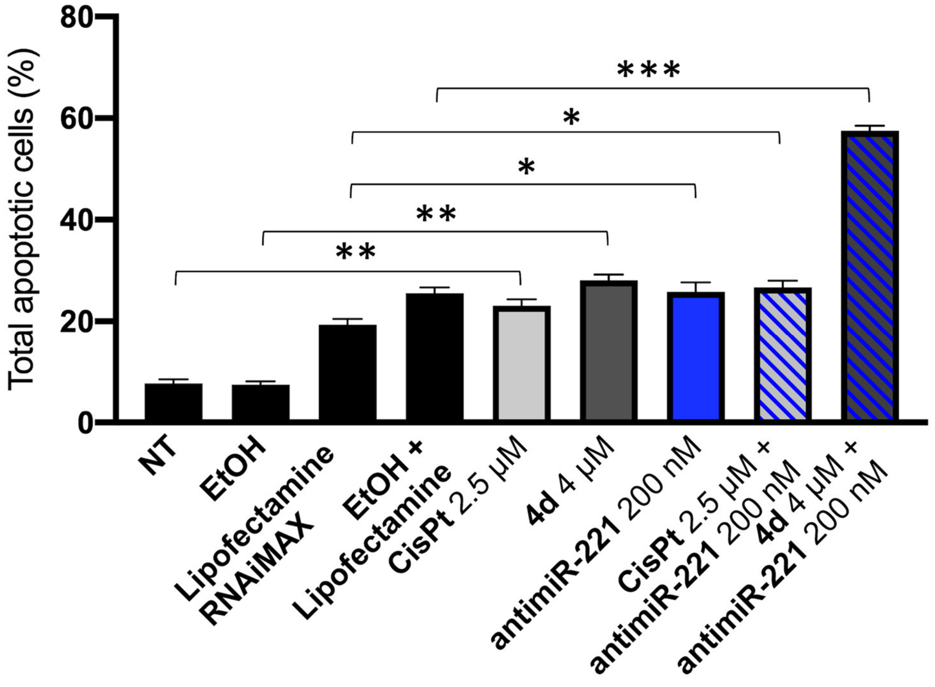

Figure S6. Total apoptotic cells percentage obtained from annexin-v staining after treatment of T98G cell line with CisPt and compound 4d in combination with antimir-221. Complete data set obtained from three different experiments, including each vehicle employed and showing comparisons evaluated for statistical analysis (ANOVA). $p < 0.05$ (*, significant), $p < 0.01$ (**; highly significant), $p < 0.001$ (***; highly significant).

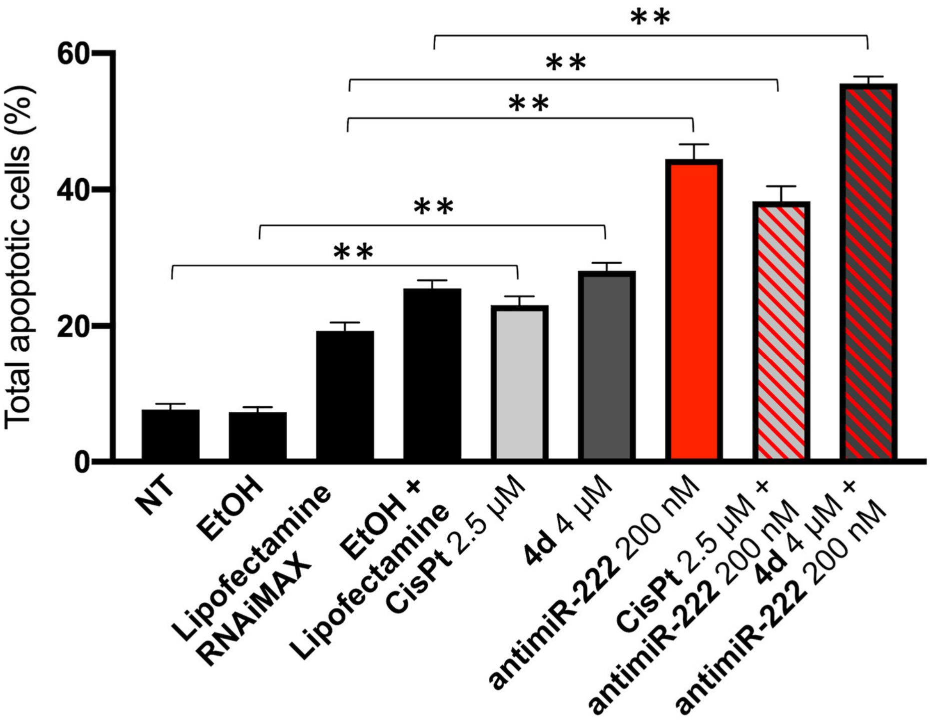

Figure S7. Total apoptotic cells percentage obtained from annexin-v staining after treatment of T98G cell line with CisPt and compound 4d in combination with antimir-222. Complete data set obtained from three different experiments, including each vehicle employed and showing comparisons evaluated for statistical analysis (ANOVA). $p < 0.01$ (**; highly significant).
